# Oxidative Stress in Malaria

**DOI:** 10.3390/ijms131216346

**Published:** 2012-12-03

**Authors:** Sandro Percário, Danilo R. Moreira, Bruno A. Q. Gomes, Michelli E. S. Ferreira, Ana Carolina M. Gonçalves, Paula S. O. C. Laurindo, Thyago C. Vilhena, Maria F. Dolabela, Michael D. Green

**Affiliations:** 1 Oxidative Stress Research Laboratory, Institute of Biological Sciences, Federal University of Para (LAPEO/ICB/UFPA) Av. Augusto Correa, 1, Guama, Belem, Para 66075-110, Brazil; E-Mails: alydan@hotmail.com (D.R.M.); brunoquadros@yahoo.com.br (B.A.Q.G.); mferreira@ufpa.br (M.E.S.F.); acmusa23@yahoo.com.br (A.C.M.G.); paula.biomedica@gmail.com (P.S.O.C.L.); tc-vilhena@hotmail.com (T.C.V.); 2 Pharmacy Faculty, Institute of Health Sciences, Federal University of Para. Av. Augusto Correa, 1, Guama, Belem, Para 66075-110, Brazil; E-Mail: fani@ufpa.br; 3 US Centers for Disease Control and Prevention, 1600 Clifton Road NE, mailstop G49, Atlanta, GA 30329, USA; E-Mail: MGreen@cdc.gov

**Keywords:** oxidative stress, malaria, free radicals, antioxidants, *Agaricus sylvaticus*, nitric oxide

## Abstract

Malaria is a significant public health problem in more than 100 countries and causes an estimated 200 million new infections every year. Despite the significant effort to eradicate this dangerous disease, lack of complete knowledge of its physiopathology compromises the success in this enterprise. In this paper we review oxidative stress mechanisms involved in the disease and discuss the potential benefits of antioxidant supplementation as an adjuvant antimalarial strategy.

## 1. Introduction

According to the World Health Organization (WHO), malaria is a significant public health problem in more than 100 countries and causes an estimated 200 million infections each year, with more than 500 thousand deaths annually. Over 90% of these deaths occur in sub-Saharan Africa, where the disease is estimated to kill one child every 30 seconds [1]. In other areas of the world, malaria causes substantial morbidity, especially in the rural areas of some countries in Asia and South America. In contrast, despite previous elimination in regions like the United States and Western Europe, the phenomenon of “imported malaria” introduced by immigrants and travelers, still contributes with sporadic cases in these regions [2].

In Brazil, a country in which malaria is endemic, the situation is equally alarming. Even with a cutback in the number of reports on malaria cases in recent years, the high risk of malaria incidence and transmission in the Amazon region persists. According to the Ministry of Health, 99.7% of malaria cases are concentrated in the Amazon region. Strengthening of the national malaria control program in 2000 has resulted in a steady decrease since 2005, according to the Annual Parasitic Incidence in the Amazon area. Although the malaria rate has decreased, resistance to drug therapy has increased, especially in patients infected with *Plasmodium falciparum*, responsible for about 20% of the cases in this region [3].

In fact, current drugs such as chloroquine [4] and artemisinin [5,6], already present resistant strains of *Plasmodium falciparum*. However, factors leading to this resistance are still not well known owing to a lack of thorough understanding on the physiopathogenic mechanisms of the disease.

Several authors have discussed the implications of free radicals through oxidative stress in the physiopathogenesis of malaria [7–18]. This involvement may be related to the pathogenic mechanisms triggered by the parasite [19], as well as free radical production [20] and antioxidant defenses [21] in host cells to abate the infection.

The role of oxidative stress during malaria infection is still unclear. Some authors suggest a protective role, whereas others claim a relation to the physiopathology of the disease [21]. However, recent studies suggest that the generation of reactive oxygen and nitrogen species (ROS and RNS) associated with oxidative stress, plays a crucial role in the development of systemic complications caused by malaria. Malaria infection induces the generation of hydroxyl radicals (OH^•^) in the liver, which most probably is the main reason for the induction of oxidative stress and apoptosis [22]. Additionally, Atamna *et al*. [23] observed that erythrocytes infected with *P. falciparum* produced OH^•^ radicals and H_2_O_2_ about twice as much compared to normal erythrocytes.

A potential source of free radical production in this disease is the host’s hemoglobin molecule, since the parasite uses this molecule as a source of amino acids for its own nutrition during the erythrocytic stage of the disease, resulting in the liberation of large amounts of circulating heme. By having Fe^2+^-associated groups, these heme groups are able to induce intravascular oxidative stress, causing changes in erythrocytes and endothelial cells and facilitating the internalization of the parasite in tissues such as the liver and brain [14].

A free radical species, which appears to be involved in this disease is nitric oxide (NO) [7–12,24,25]. However, its role is still controversial. Some researchers claim that cerebral malaria is probably an unfortunate consequence of high amounts of NO production to promote the death of the parasites [26,27] while others support the idea that cerebral malaria results from a low bioavailability of this compound [28].

Additionally, host-parasite interactions are quite complex and promote constant changes in the delicate balance between pro-oxidant and antioxidant molecules since the host and parasite are capable of producing both. Nevertheless, even anti-malarial drug therapy constitutes a source of oxidation, as many drugs such as chloroquine, primaquine and derivatives of artemisinin are inducers of free radical production [29–31].

This review endeavors to present the oxidative stress mechanisms in malaria as well as discuss the potential benefits of antioxidant supplementation therapy as an adjunct to anti-malarial treatment.

## 2. Oxidative Alterations in the Host Induced by *Plasmodium*

In response to infection caused by *Plasmodium* parasites, the natural host defense mechanism is activated with involvement of phagocytes (macrophages and neutrophils). These, in turn, generate large amounts of ROS and RNS, causing an imbalance between the formation of oxidizing species and the activity of antioxidants. This imbalance is what triggers oxidative stress, which is an important mechanism of human hosts in response to infections and, in the case of malaria, can lead to the death of the parasites.

*In vitro* studies have demonstrated the ability of oxidative stress to promote the killing of parasites. Incubation of *Plasmodium yoelii* species in the presence of glucose and glucose oxidase generated H_2_O_2_, a reactive oxygen species, capable of killing the parasite. Likewise, when incubated in the presence of xanthine and xanthine oxidase, it generated free radical superoxide (O_2_^•−^) and a subsequent burst of other oxidative products, with consequent destruction of parasites [32].

Furthermore, oxidative stress markers in infected humans and rats are found in high levels compared to uninfected controls [21,22,33–35]. In such cases oxidative stress seems to result from increased production of free radicals, a fact suggested by increased malondialdehyde (MDA), an important lipid peroxidation marker, and not from a decrease in levels of antioxidants, reinforcing the suggestion that oxidative stress is an important mechanism in parasite infection [24].

Recent studies suggest that oxidative stress can take part in the pathogenesis of thrombocytopenia associated to malaria. Erel *et al*. [36] showed that the number of platelets and the activities of antioxidant enzymes—superoxide dismutase (SOD) and glutathione peroxidase (GSH-Px)—in patients with *vivax* malaria were reduced while lipid peroxidation of platelets (estimated by measuring the MDA), was elevated in infected individuals, suggesting a negative correlation between platelet count and platelet level of lipid peroxidation. These data suggest that oxidative stress occupies an important role in the genesis of thrombocytopenia present in malaria through loss of elasticity of membranes and by increasing brittleness and causing dysfunction in receptors, resulting in considerable functional impairiment of thrombocytes.

Indeed, the importance of these radical species in the genesis of rheological changes in malaria patients has led us to believe that one should avoid iron replacement in infected individuals, despite the degree of anemia observed by the erythrocytes count [36,37].

Along with the synthesis of radical species, organisms have developed different antioxidant defense mechanisms in response to increased oxidative stress. In fact, antioxidant defense is a natural physiological mechanism of organisms against damage caused by free radicals and it depends on the consumption of cellular and systemic antioxidant reserves. Endogenous synthesis of these antioxidant compounds typically consists of three interdependent systems: enzymatic, small molecules and metal chelation, which retards or prevents oxidation of biomolecules. The antioxidant defense system also avoids oxidative species generation by scavenging or by free radicals reduction, which by self-oxidation form less reactive compounds [38].

Although several antioxidant enzymes are important in the defense system, the most important include GSH-Px, catalase and SOD. These enzymes act directly on some free radicals, making them less reactive. However, they are not able to act on the highly reactive free radicals that are chiefly responsible for oxidative pathological processes such as hydroxyl and perhydryl radicals or peroxynitrite.

As a result, our body uses small molecules that reduce the reactivity of various reactive radicals as an auxiliary antioxidant defense system. This group contains a large number of molecules, such as vitamins A, C and E, beta-carotene, uric acid and reduced glutathione molecule (GSH). In addition, our organism has proteins that bind to transition metals preventing them from catalyzing the Fenton and Haber-Weiss reactions, important sources of reactive species production. These metal chelators include ferritin, transferrin and lactoferrin (chelating iron), ceruloplasmin and albumin (copper chelators) and metallothioneins having thiol groups capable of binding several heavy metals [39].

Among the antioxidant molecules, the GSH molecule stands out as being the most powerful protector of eukaryotic cells in the host defense against oxidative stress, acting upon several different mechanisms [40]. In parallel, the secretion of tumor necrosis factor-alpha (TNF-α) appears to induce oxidative stress through modulation of GSH metabolism, playing an important role in malaria physiopathogenesis. In studies with rats, the administration of TNF-α induced decreased GSH levels, whereas in CD4+ and CD8+ splenic T cells, a significant increase occurred in oxidized glutathione (GSSG) [41], thus both behaviors suggest oxidative stress increase. Several authors have reported decreased GSH in malaria patients [40,42].

However, besides GSH, lower levels of various antioxidants are found in malaria patients caused by *Plasmodium vivax*. These are: antioxidant enzymes and glutathione *S*-transferase (GST) [21,43], catalase, GSH-Px, SOD [11,42], NADPH-methemoglobin reductase [11]; heavy metal chelators desferrioxamine, salicylaldehyde isonicotinoyl [44] and ferritin [42]; small molecules such as vitamins A, E, C [45–47]; the pro-vitamins α- and β-carotene, lycopene, lutein and zeaxanthin [45], among others.

Similarly, *Plasmodium falciparum* malaria patients presented lower levels of antioxidants, such as ascorbate, which correlated with disease severity, in contrast to elevated levels of urate and ceruloplasmin [48]. Accordingly, the increase in levels of urate may indicate the presence of ischemia-reperfusion syndrome (IRS) responsible for free radical production in ischemic conditions or even in hypoxia [49] due to parasite-induced hemolysis and cytoadherence.

Likewise, decreased GST activity is directly related to the severity of the parasitemia, since the production of this enzyme reduces complications of malaria and occurrence of severe malaria [21,43]. In this sense, the assessment of GST levels, lipid peroxidation and catalase, may be considered as potential biochemical markers of disease severity.

In children, all acute phase proteins (APP) are useful markers of the type and severity of inflammation in malaria, since all APP, except for α1-antichymotrypsin, were significantly correlated with splenomegaly, while α1-acid glycoprotein (AGP) and C-reactive protein (CRP) indicated chronic inflammation [50]. Likewise, concentrations of albumin, apolipoprotein A1 (apoA1), transferrin, zinc, vitamin A, immunoglobulins G and M, interleukin 10 (IL-10), tumor necrosis factor-alpha (TNF-α) and interferon-gamma (IFN-γ) were verified. Children with malaria had decreased levels of apoA1 and albumin, but high levels of IL-10 when compared to children without malaria. All antioxidants studied showed lower levels in patients with the disease [51].

In another study, mice infected with *Plasmodium berghei* showed significantly increased lactate and alanine concentrations in the final stage of cerebral malaria, additionally, glutamine and essential amino acids levels were slightly elevated in the brain [52].

Mice infected with *P. vinckei vinckei* exhibit erythrocytic protection against reactive oxygen species by the enzymes superoxide dismutase, catalase, glutathione peroxidase, glutathione reductase, NADPH and NADH-methemoglobin reductase in red blood cells [34].

In cultured endothelial cells incubated with heme, an aggravation of oxidative stress mediated by polymorphonuclear leukocytes was observed, which may probably be reversed by heme-oxygenase and ferritin mRNA induction, additionally to the administration of antioxidants such as catalase, reduced glutathione and superoxide dismutase [42].

Another possibility of damage reversion consists in the administration of iron chelators, which inhibits the growth of *P. falciparum in vitro*, such as hydrophilic desferrioxamine (DFO). Related factors to the use of these substances showed greater efficiency of anti-malarial drug action [44].

*Plasmodium berghei* infection in mice induces liver injury, increases mRNA expression of interleukin-12 (IL-12), protein 40 (p40) as well as IFN-γ, interleukin-4 (IL-4) and IL-10, with consequent increase in NO synthesis. Treatment with anti-IL-12 provides an indirect reduction of free radical production, thus prolonging survival, reducing liver damage and weight loss, but without alteration of the parasitemia [53].

A study conducted with 273 children between the ages of 1–10 with acute uncomplicated *P. falciparum* malaria in Kampala, Uganda, verified the antioxidant status in the pathogenesis of the disease. Children with acute malaria had low antioxidant plasma concentrations. On the other hand, in children with higher plasma lycopene levels, there was fast parasitemia clearance [45].

In addition to lycopene, other antioxidant substances are known to act as an adjunct in drug therapy such as riboflavin, a reducing agent that acts in the parasite food vacuole and hemozoin formation [54], which, as allicin, is an inhibitor of cysteine protease found in garlic extracts and acts by inhibiting circumsporozoite protein (CSP) processing, essential for the invasion of host cells [55].

Oxidative stress is commonly observed to arise from five sources during disease physio-pathogeny: 1, Inflammatory process initiated in the host in response to infection; 2, transition metal catalysis, since in feeding on hemoglobin, the parasite releases significant amounts of free iron; 3, the occurrence of ischemia-reperfusion syndrome, resulting from cytoadherence processes and anemia triggered by infection; 4, direct reactive species production by the parasite; and 5, action of antimalarial drugs.

### 2.1. Oxidative Stress as Host Defense Mechanism against *Plasmodium* Infection

It has been shown that oxidative stress is related to a protective role in malaria patients, as possible agents capable of destroying the *Plasmodium*. Thus, H_2_O_2_ and O_2_^•−^ can operate independently as cytotoxic agents or form other toxic molecules, including radical OH^•^, hypochlorous acid (HOCl) and peroxynitrite (ONOO^−^) in the presence of NO [22].

The increased production of ROS by phagocytes, as part of the host defense, is a primary event. ROS generated by macrophages are known as non-specific effectors molecules in the host’s defense arsenal, which can contribute to oxidative damage in the parasite as well as parasitized erythrocytes, once ROS are able to diffuse through the membrane of red blood cells [56]. Likewise, neutrophils secrete proteolytic enzymes and ROS, which in low concentrations can trigger apoptosis of endothelial cells and necrosis in high concentrations [57].

In fact, it is generally accepted that ROS, including O_2_^•−^ and ONOO^−^, can destroy the parasite intraerythrocytically [35]. Therefore, severe malaria caused by *Plasmodium falciparum* is associated with activation of neutrophils and monocytes, elevated levels of cytokines and endothelial injury. Thus, activated neutrophils and their secreted products can exert not only anti-parasitic, but also endothelial injury, which can lead to organic failure in severe malaria [57].

The inflammatory process mediated by effector immune response mechanisms is required as the homeostasis of the organism is modified. However, in some cases, such as in malaria, these mechanisms are not sufficient to eliminate some *Plasmodium* species and to some extent, may act in a manner that further harms the host cell.

With regard to malaria, the immune mechanisms are not fully understood yet, but it is known that the cytokine profile, which is proven effective in tackling the infection, is the mechanism that involves cellular response by helper T lymphocytes (Th) 1, with prevalence of TNF-α, IFN and IL-12. On the other hand, the Th2 profile (IL-4, IL-5, IL-10 and Transforming Growth Factor beta—TGF-β) are considered to aggravate severe conditions of the disease. However, there is no consensus on these profiles [56,57], because different cellular responses act efficiently in different stages of parasite elimination: the first, essential at the initial process of infection, allows the host to mount an adaptive immune response against the parasite, but on becoming exaggerated or chronic, may favor severe anemia processes; the second promotes the clearance of the initial response (Th1) to be replaced by the adaptive action response. Obviously, if such responses are not properly activated, the parasite will tend to resist host defense [58].

Nevertheless, we know the importance of ROS/RNS participation in the parasitemia elimination process as the main mechanism through which most anti-malaria drugs act. These substances are also known to regulate immune responses by stimulating or inhibiting production of a certain cytokine, transcription factor and even regulating cell death processes [59].

Apoptosis is a natural process of cell death induced by intracellular mechanisms without the release of intracellular contents in the extracellular matrix. Such a mechanism is particularly interesting, since toxic substances from intracellular apoptotic cells do not harm surrounding cells, unlike necrosis. Cytochrome c, a mitochondrial protein, is among the substances known to regulate this process, which under normal conditions, linked to phospholipid (cardiolipin), participates in the electron transport chain [60]. However, under stressful conditions, cardiolipin is oxidized, releasing cytochrome c, which upon reaching the cytosol, forms complexes with activating proteins of the apoptotic process.

The oxidation process of fatty acids, termed lipid peroxidation, appears to originate from the cardiolipin oxidation in the apoptosis context. Dey *et al*. [61] attribute the hydroxyl radical with the function of triggering the process in hepatocytes.

Further to lipid peroxidation, it is known that NO can play a key role in eliminating the parasite. According to Jaramil *et al*. [62], despite the contribution of NO as a free radical in the destruction of *Plasmodium*, too much NO causes immune-suppression and leads to the development of cerebral malaria. Some parasitic molecules are well known for inducing NO, but there is a direct contribution of malarial pigment—hemozoin, which, associated with the IFN mediator of mRNA synthesis by the enzyme nitric oxide synthase (iNOS), is a potent inducer of NO generation in macrophages via extracellular signal regulated by kinase (ERK) and nuclear factor κB (NF-κB). It is also known that at the hepatic stage, defense mechanisms are strictly related to the production of IFN by Natural Killer cells (NK), with subsequent NO synthesis [63].

It has also been observed that in addition to inducing NO, hemozoin is responsible for macrophage activation through mechanisms partially dependent on NO [15] and, according to Brinkmann *et al*. [64], on other ROS/RNS such as the superoxide radical and hydrogen peroxide. Similarly, increased levels of iNOS in human monocytes are not associated with the worsening of malaria in patients infected with *P. falciparum*[65].

Syarifah *et al*. [66] studying mice susceptible and resistant to cerebral malaria infected with *Plasmodium berghei*, observed that the expression of cytokines (except for IL-4 and RANTES) as well as the expression of NO tested on mice spleen cells was enhanced in cells of resistant animals when compared to cells of susceptible animals. It is important to emphasize the high production of TNF-α in resistant mice, suggesting that activation of macrophages is significantly higher in these animals. Corroborating these findings, Kumaratilake *et al*. [67], using radiometric assays that quantify the antiplasmodial effect of phagocytes, verified in humans infected by *P. falciparum* that TNF-α increased the release of toxic reactive oxygen intermediates on neutrophils, which contributed to the elimination of the parasite.

Additionally, it has been demonstrated that the free heme can stimulate both neutrophil migration and ROS/RNS production by a G protein-coupled receptor, more specifically the inhibitory Gα receptor, which in turn activates protein kinase C, increasing the inflammatory response [68] and delaying apoptosis [59], thereby most probably contributing to the immune-suppression induced by malaria [69].

Among several cytokines already studied, an increase in the Granulocyte-Macrophage Colony-Stimulating Factor (GM-CSF) appears to correlate with the reduction of parasitemia and, in this context, with oxidative stress. GM-CSF is a cytokine with stimulatory action on granulocytes and macrophages and promotes an increase in the number and activity of these cells, acting effectively in cellular immunity against malaria. Several studies have shown that previous administration of GM-CSF, alone or associated with other factors, protected experimental animals from infection by various parasites [70,71].

Similarly, animals deficient in GM-CSF synthesis had an impaired immune response against parasite infection [72]. There seems to be a relation between GM-CSF and oxidative stress: GM-CSF and IL-4 receptors may be modified by lipid peroxidation products originating from hemozoin released by rupture of parasitized erythrocytes, such as 4-hydroxynonenal (4-HNE). GM-CSF and IL-4 are stimulators of the differentiation of hemozoin-loaded monocytes in dendritic cells. This differentiation can be inhibited by the 4-HNE and play an important role in immune suppression present in malaria [73].

Moreover, this mechanism appears to be associated with the expression of peroxisome proliferator-activated receptor gamma (PPAR-γ) in immature dendritic cells loaded with hemozoin following administration of (15) *S*-hydroxyeicosatetraenoic acid—a PPARγ ligand produced by hemozoin via peroxidation induced by the heme—which inhibits the differentiation process of these cells [74].

There is also a relationship between GM-CSF and NO, as shown by Kaur *et al*. [71], demonstrating that pre-treatment with GM-CSF and methionine encephalin (TGG) protects mice from malaria. However, when animals were pre-treated with inhibitors of inducible form and both forms of NOS, the mortality rate of the animals increased significantly, suggesting that the protection exerted by GM-CSF/TGG was, at least partially, due to NO.

#### 2.1.1. The Role of NO in the Physiopathology of Malaria

NO is one of the smallest and simplest biosynthesized molecules [75]. It is an inorganic, colorless, free radical gas, with seven nitrogen and eight oxygen electrons [76] and presents a sometimes beneficial, sometimes harmful dubious role in the body [77]. It has been shown to be involved in many important biological functions, such as vasodilatation, blood pressure regulation, inflammation and unspecific immunity, neuronal plasticity, as well as to act as a neurotransmitter in the brain and peripheral nervous system. This molecule is also capable of lysing tumor cells [78,79].

It is produced from the l-arginine substrate by nitric oxide synthase (NOS). Nitric oxide is produced in significant quantities during the inflammatory response by macrophages and other immune cells, which express the inducible isoform of nitric oxide synthase (iNOS). Nitric oxide produced in these circumstances causes lethal oxidative damage to target cells, such as bacteria and other micro-organisms [80].

Despite being an important cytotoxic mediator of immune activated cells, capable of destroying pathogens and tumor cells, NO is potentially toxic, mainly in situations of oxidative stress, which leads to the generation of reactive oxygen intermediates and an antioxidant system deficiency [77].

Nitric oxide plays an important role in physiological functions and, consequently, many diseases may be related to a high or low NO level in the body. The role of nitric oxide in blood-stage malaria remains unknown.

Many authors believe that NO is not capable of killing parasites at in the blood-stage of malaria. They have reached this conclusion by comparing serum nitric oxide levels in *Plasmodium* infected mice and humans against control levels in mice and human individuals. These studies conclude that nitrite and nitrate levels (NN—stable metabolites of NO) in infected individuals do not differ from control levels, and even with an increased NN serum concentration during the blood-stage of malaria, parasitemia is not different compared to control values [26,35,81–87].

Sobolewski *et al*. [35] also questioned the role of NO in the progression of malaria parasitemia, stating that intraerythrocytic *Plasmodia* are protected from reactive oxygen species by hemoglobin, occurring naturally in high concentrations within red blood cells (RBC), requiring levels of reactive oxygen species that surpass hemoglobin protection. In their experiments, NO was not able to fight the parasite until levels close to saturation. Other studies with iNOS−/− knockout mice found parasitemia levels similar to that of control animals [82,88].

However, some researchers like Stevenson *et al*. [58] and Böhlke [89] suggest that NO has a protective role against blood-stage malaria and claim that NO is an essential factor for malaria resolution by *P. Falciparum*. They believe that elevated serum nitric oxide levels are toxic to the parasite [90].

In our personal experience, despite decreasing parasitemia in infected mice by *P. berghei*, inhibition of NOS enzymes favors the development of severe forms of the disease, notably cerebral malaria, suggesting that NO is a protective factor preventing aggravation of the disease [91].

The chief answer to this impasse is that NO can act both directly and indirectly on malaria resolution. It may have a direct action as a parasiticide explained by the action of peroxynitrite, resulting from the reaction of NO with O_2_^•−^. Although indirectly, nitric oxide contributes to parasiticidal activity. This can occur through increased immune activity, absence of IL-4, the relationship between NO and GM-CSF/PPAR-γ, or the relationship between NO and increased ROS/RNS.

Nevertheless, we also agree that NO has a parasiticidal role in malaria, since high serum levels of it favor parasitemia resolution without harming the host. This gas, previously seen as a toxic agent, is considered today as a way to resolve this disease, since it may directly act on the inflammatory process and indirectly enable the necessary cytokines to activate the immune system.

NOS enzymes are dimeric flavoproteins containing tetrahydrobiopterin homologous to cytochrome P450. There is a classification type that differentiates enzymes according to their physiological function, and names them by inducible NOS (iNOS) and constitutive NOS (cNOS). The other classification type for NOS enzymes is based on the type of cell in which they are present. These are named: NOS1 (present in the cytosol of neurons) [92], NOS2 (present in immune system cells) [93,94], and NOS3 (present in endothelial cells) [95].

Studies indicate that NO produced by iNOS activation plays an important role in killing several pathogens, including *Plasmodium*[96]. However, recent studies in animal models have shown that NOS deficiency or inhibition is not enough for protection against *P. berghei*[26,82,86,97] but hemozoin induces NO formation via iNOS [20] and the increase in monocyte count may be associated with high levels of mRNA of this enzyme [65].

In contrast, NO produced by endothelial NOS on all tissues acts as blood flow regulator, causing vasodilatation, preventing platelet aggregation and inhibiting adhesion of lymphocytes and monocytes to to endothelium [77,79], which prevents local ischemia. These effects are crucial in preventing cerebral malaria, and the administration of exogenous NO, or substances that release NO, has been investigated as adjunctive treatment for malaria, with great results in improving microcirculation, reducing brain inflammation and protecting the blood-brain barrier [24,98–101], thus decreasing oxidative stress. Exogenous NO is also indicated in the prophylaxis of lung damage of malaria patients [102].

Infection with the malaria parasite in the *Anopheles stephensi* mosquito induces the expression of nitric oxide synthase. This induction results in the synthesis of inflammatory levels of NO in the host circulation, causing an impact on parasite development. It has also been demonstrated that glycosylphosphatidylinositol can induce the expression of NOS [103].

An important fact is that livers from immunized mice express mRNA for iNOS between 12 and 24 h after the arrival of sporozoites in the liver, and these animals, even when treated with a NOS inhibitor, are completely protected against the parasite at the erythrocyte stage of malaria [104].

Other studies have correlated the incidence of malaria to changes in the promoters of genes encoding the enzymes iNOS and G6PD [105].

#### 2.1.2. Hemolysis as an Oxidative Stress Induction Factor in Malaria

During the erythrocytic phase of malaria, red blood cell lysis and the release of hemozoin occurs, which consists primarily of ferriprotoporphyrin IX dimers and monomers (FP) and methemoglobin in plasmodial proteins. Hemozoin is able to induce cytokine release (TNF-α and IL-1) through cells of the monocyte/macrophages system. Free heme is a powerful free radical generator, which can cause serious molecular damage to both host and parasite; the heme group contains Fe^2+^ atoms that can catalyze Fenton and Haber-Weiss reactions, generating free radicals. This is why certain drugs, such as chloroquine, have an active mechanism to prevent hemozoin formation, promoting accumulation of free heme. Thus, chloroquine increases the availability of intracellular heme by disrupting the plasma membrane structure and increasing oxidative stress in *Plasmodium*.

As a result of oxidative stress, lipid peroxidation occurs, promoting functional and structural changes of the plasma membrane that lead to hemolysis, which has always been linked to increased levels of thiobarbituric acid reactive substances (TBARS), markers of lipid peroxidation. The high levels of lipid peroxidation products, such as TBARS, have already been seen in erythrocytes parasitized by *P. falciparum*, *P. vinckei*, *P. berghei*, *and P. chabaudi*[56].

Hemolysis or extensive cell damage can lead to increased concentrations of free heme, causing oxidative stress and inflammation. Whereas heme induces neutrophil chemotaxis, Porto *et al*. [68] reported that several heme analogs are able to induce neutrophil migration *in vitro* and *in vivo*, and that mesoporphyrins, molecules lacking vinyl groups in their rings, were not chemotactic for neutrophils and selectively inhibited heme-induced migration. The authors conclude that heme activates neutrophils through chemoattractant signaling and that mesoporphyrins may be important in the treatment of inflammatory consequences such as bleeding and hemolytic disorders.

Additionally, the cellular response to hemozoin entails cytokine release [15,105–107] and generation of reactive oxygen species such as NO [106]. Research on oxidative stress induced by hemozoin/heme is exciting the scientific community because supports the development of new drugs, as is the case with the potential antimalarial [(aryl)arysulfanylmethyl] Pyridine (AASMP) [108].

#### 2.1.3. Oxidative Stress and the Membrane of Infected-Erythrocyte

During the development of the blood esquizogeny, *P. falciparum* trophozoites increase the viscosity of red blood cells by causing changes in the parasitized cell surface permitting its adhesion to the endothelial wall of capillaries, which seems to be a defense mechanism of the parasite, preventing the passage of parasitized red blood cells through the spleen and their consequent destruction [109]. However, the increased viscosity of the cells appears to be primarily responsible for the blocking of blood vessels, especially of kidney capillaries, pulmonary capillaries and brain capillaries, and cerebral malaria is the most common reason for coma and death in infected children [110,111].

Among the changes that take place on the surface of red blood cells is the phenomenon of lipid peroxidation. In this sense, the parasitized erythrocytes are known to contain large amounts of monohydroxy derivatives of polyenoic fatty acids (OH-PUFA) in their lipids, suggesting the occurrence of lipid peroxidation due to the release of heme iron from non-enzymatic breakdown [112]. One of the common OH-PUFA found and already described as toxic is the 12- and 15-hydroxy-arachidonic acid (HETE). It is known that the concentrations of OH-PUFA increase according to the evolutionary stage of the parasite. However, low concentrations of HETE were found after phagocytosis of parasitized RBCs, suggesting that other lipid peroxidation products also may play a key role in this process [112].

Additionally, oxidative changes in *P. falciparum*-infected red blood cells seem to be associated with the accelerated aging of these cells and contribute to the development of anemia presented by these subjects [10]. The development of anemia can promote changes in the circulatory physiology, leading to the existence of moments of hypoxia alternating with the maintenance of tissue oxygenation at basal levels, favoring the participation of ischemia and reperfusion syndrome (IRS) responsible for an additional production of free radicals [39].

Indeed, increased lipid peroxidation and oxidative stress reported in human malaria can affect the membrane of infected erythrocytes, also promoting the reduction of the deformity of these cells, which has been linked to increased mortality of adults and children with malaria. The deleterious consequences of increased cell rigidity include microcirculatory obstruction (exacerbating tissue hypoperfusion) and cell stiffness with subsequent removal by the spleen, which increases anemia [11].

However, in *P. falciparum* infections, red blood cells infected by mature trophozoites and schizonts are sequestered in the capillaries of various organs, which prevents their removal by the spleen. The adhesion of these cells to the vascular endothelium (cytoadherence phenomenon) can be related to a large number of antigenic variants encoded on the parasite’s surface that bind to endothelial receptors [113].

This cytoadherence phenomenon is mediated by parasite proteins expressed via stimulation of the *var* gene on the surface of infected red blood cells, such as *P. falciparum* erythrocyte membrane protein 1 (PfEMP1). The expression of such proteins allows these parasitized erythrocytes to connect to different host molecules located in the vascular endothelium, such as intercellular adhesion molecule type 1 (ICAM-1), platelet endothelial cell adhesion molecule (PECAM), vascular cell adhesion molecule (VECAM), hyaluronic acid, heparin sulfate and others, interrupting blood flow and causing the impairment of tissues irrigated by clogged vessels [113,114].

Other parasite proteins that promote increased membrane stiffness of infected erythrocytes are histidine-rich protein associated with deformity (KAHRP) and 3 erythrocyte membrane protein of *P. falciparum* (PfEMP3). Studies have shown that in the absence of both proteins, membranes showed low levels of rigidity, and that KAHRP had a greater effect on the stiffness than PfEMP3, suggesting that these parasite proteins contribute to the rigidity of red blood cells [115].

#### 2.1.4. Ischemia and Reperfusion Syndrome as Oxidative Stress Induction Factors in Malaria

IRS occurs in malaria during sequestration of parasitized erythrocytes in certain tissues as a result of disrupting large amounts of erythrocytes promoted by *Plasmodium* during malarial paroxysm and of cytoadherence of erythrocytes to blood vessels.

In this syndrome, restriction of blood flow leads to O_2_ concentrations lower than normal [39], resulting in the uncoupling of mitochondrial oxidative phosphorylation leading to a decrease in adenosine triphosphate (ATP) production. This is followed by the consumption of stored cellular ATP, which is further degraded to adenosine diphosphate (ADP) and adenosine monophosphate (AMP) and finally to adenosine. Should irrigation be restored at this stage, adenosine may be re-phosphorylated to ATP. However, if hypoxia persists, adenosine will be irreversibly metabolized to inosine and hypoxanthine. During ischemia, ATP shortage would cause failure of sodium-potassium dependent ATPase, with consequent accumulation of intracellular Na^+^ and extracellular K^+^. These alterations could trigger the occurrence of cellular edema by causing increased osmotic pressure and cytoplasmic decompartmentalization.

The phenomenon of cytoplasmic decompartmentalization can be seen as a cellular distress condition, in which ions kept in watertight compartments flood into the cytoplasm, causing activation of enzymes that should be inactive, triggering communication processes at the intracellular level, or promoting free radical production. In this mechanism, Ca^2+^ ions and ferrous ions (Fe^2+^) are quite important. The free Ca^2+^ in the cytosol activates the protease calpain, which in turn promotes the breakage of a peptide bond of the enzyme xanthine dehydrogenase (XD), changing its activity to xanthine oxidase (XO).

Unlike XD, XO needs oxygen to convert hypoxanthine into xanthine. Thus, during ischemia three crucial phenomena occur with merely preparatory effects: The production of hypoxanthine from ATP, the conversion of XD in XO, and cytoplasmic decompartmentalization (which also releases iron from ferritin into the cytoplasm).

On the other hand, with reperfusion, the renewed oxygen supply catalyzes the metabolism of hypoxanthine by XO. This reaction leads to the production of xanthine, uric acid, O_2_^•−^ and H_2_O_2_. It should be noted that ROS and RNS production only occurs during reperfusion. In turn with the ROS/RNS produced in reperfusion, O_2_^•−^ and H_2_O_2_ can react together in the presence of iron (Haber-Weiss reaction) to yield the OH^•^ radical and trigger the magnification and propagation of oxidative damage.

## 3. Oxidative Changes in *Plasmodium*

### 3.1. Production of Reactive Species by the Parasite

Besides host ROS/RNS production in response to infection, the parasite itself is capable of producing free radicals, which in turn interfere with the biochemistry of red blood cells and may promote or facilitate the internalization of the parasite in hepatocytes and RBC.

Despite scarce exploration in the scientific literature, aerobic membrane transport mechanisms are a major source of free radical and ROS/RNS generation in *Plasmodium*. Accordingly, a recent study found that the absence of NADPH-oxidase expression, an important enzyme in the synthesis of free radicals by macrophages, caused no differences in the progression of parasitemia in knockout mice for any of the *Plasmodium* species tested *(P. yoelii*, *P. chabaudi* K562, *P. berguei* ANKA, *P. berguei* K173 and *P. vinckei vinckei)*. These findings led the authors to believe that free radical production increased as a result of infection and not from the respiratory burst of phagocytes, possibly due to production by the parasite itself [19].

Another factor that reinforces this possibility lies in the complexity and variety of antioxidant mechanisms developed by these parasites.

### 3.2. Antioxidant Defense Mechanisms in the Parasite

*Plasmodium* parasites are subjected to high levels of oxidative stress during development in host cells, so that their ability to defend themselves against this aggression is critical to their survival. As a result, these parasites have developed several antioxidant defense mechanisms.

Studies of gene expression during the erythrocytic phase of infection by *Plasmodia* have determined that at the early stage a continuous cascade of gene expression takes place, and at least five different proteins with antioxidant properties are expressed in these conditions [116].

Additionally, to compensate for the oxidative stress suffered, *Plasmodium* reduces its own production of reactive oxygen species and adapts new mechanisms to prevent oxidative damage arising from the host. The apicoplast is one such mechanisms; it is a symbiotic intracellular organelle located near the mitochondria which seems to synthesize lipoic acid, a potent antioxidant used by the parasite as a defense. Most probably this organelle was incorporated as an evolutionary adaptation of the parasite, since this organelle is also present as a symbiont in red algae [117].

Moreover, in most *Plasmodium* cells, the redox homeostasis seems to be based on the synthesis of reduced glutathione and thioredoxin system (Trx)/thioredoxin reductase (TrxR). The reduction of oxidized glutathione (GSSG) can be supported by the high proportion of the TrxR/Trx system in glutathione reductase-deficient cells, which may be important for certain stages of the parasite [11,118].

The glutathione and thioredoxin redox systems represent two powerful means to detoxify reactive oxygen species in *Plasmodium falciparum* and they are efficient systems to prevent parasite development inside the host cells [119]. Additionally, an enzyme peroxiredoxin associated with chromatin in *P. falciparum* has been identified, which makes use of thioredoxin and glutaredoxin as reducing agents, thereby conferring protection to the parasite against the oxidative insult imposed by the host [120].

The TrxR, an enzyme involved in the maintenance of redox homeostasis and antioxidant defense, is essential for the erythrocytic stages of *P. falciparum*[121]. The disruption of the parasite antioxidant system is a feasible way of interfering with its development during erythrocytic schizogony [122].

Furthermore, glutaredoxin-1, thioredoxin-1 and plasmoredoxin are able to efficiently catalyze protein deglutathionylation, a widely distributed important mechanism of posttranslational modification of thiol groups with glutathione which functions as a intracellular redox signaling regulating device [123].

In fact, Campanele [124] found that *P. falciparum* proteins interact with ferriprotoporphyrin IX, and that thioredoxin reductase appears to be much more sensitive to inhibition by FP than glutaredoxin. However, the parasite’s glutathione reductase proves to be more resistant to being reduced by FP.

Mashima *et al*. [125] has verified that the histidine-rich protein-2 complex from *Plasmodium falciparum* (P*f*HRP2) connected to ferriprotoporphyrin IX has antioxidant properties beneficial to the parasite, which may not have been previously recognized by host antioxidants. In neutral pH, P*f*HRP2 modulates the redox activity of ferriprotoporphyrin IX, protecting ascorbate from degradation induced by FP and transition metals and ensuring release inhibition of intermediates of the lipid hydroperoxide metabolism.

Another important antioxidant molecule produced by *Plasmodia* is glutathione reductase. According to Stocker *et al*. [34], the activity of glutathione reductase, evaluated by blood GSH levels, was enhanced in malaria patients only with high levels of infected RBC, probably indicating that a significant portion of the increase in GSH was associated with the production by the parasite itself. Kehr *et al*. [120] identified sites of cellular compartmentalization for this enzyme in *Plasmodia*.

Moreover, reduced production of glutathione in *Plasmodia* is not only involved in maintaining an adequate intracellular redox environment protecting cells against oxidative stress, but has also shown to be linked to unpolymerized FP degradation, thus implicating an increase in chloroquine resistance. In a study by Meierjohann *et al*. [126], the authors verified possible differences in the GSH metabolism regulation of chloroquine-sensitive and chloroquine-resistant species of *Plasmodium falciparum*, using a γ-glutamylcysteine synthetase inhibitor and a glutathione reductase inhibitor. It was observed that *P. falciparum* Dd2 species appeared to be more capable of maintaining intracellular GSH than the *P. falciparum* 3D7 species, showing different susceptibility to oxidative stress. Likewise, resistance of *P. vinckei vinckei* to artemisinin appeared to be mediated by GSH action [127].

In Müller’s opinion [128], the *Plasmodia* defense center comprises superoxide dismutase and thioredoxin-dependent peroxidase, which, however, also needs catalase and glutathione peroxidase. The vital importance of the thioredoxin redox cycle formed by NADPH, thioredoxin reductase and thioredoxin, is essential for *P. falciparum* survival. The parasite also contains a complete functional system and GSH of low molecular weight as important intracellular tioredox protection and as a cofactor for the redox activity of glutathione S-transferase and glutaredoxin enzymes.

Another antioxidant molecule described in these parasites is vitamin B6, an essential cofactor in more than 100 enzymatic reactions. *Plasmodium falciparum* possesses a functional vitamin B6 uptake system, which is required as it is subjected to stress. This parasite expresses proteins PdX1 and PdX2, essential for the biosynthesis of this vitamin. Both plasmodial proteins act together in glutaminase activity. However, in order to be an active cofactor, vitamin B6 needs to be phosphorylated by pyridoxine kinase [129]. Therefore, inhibition of vitamin B6 synthesis mechanisms may be a potential pharmacological target to be explored.

## 4. Oxidative Changes in Vectors

Another point to be discussed about malaria is the role of *Anopheles* mosquitoes in the transmission of the disease and what instruments these vectors use to restrain the advance of evolutive phases of the parasite responsible for the onset of sporozoites, thereby checking a potential correlation with oxidative stress and antioxidant defenses.

It has been a prevailing thought that the development and maintenance of certain *Plasmodium* species in *Anopheles* mosquitoes is closely linked to vector susceptibility. This way, the evolution of the parasite to the sporozoite form must trespass the immunological barrier of the mosquito. Some strains of *Anopheles gambiae* are known to resist the parasitic evolution process mainly by oxidatively converting tyrosine to melanin, thereby aggregating it around the parasite. This defense mechanism is highly associated with the deficiency of the antioxidant machinery by these strains. For instance, initial steady-state catalase mRNA expression levels were found, but were not able to influence increased H_2_O_2_ production [130].

Great attempts have been made to discover the role of the NO molecule in eliminating the malaria parasite. This molecule exerts, at least in the mosquito, a protective role reducing parasitemia. In addition, some molecules of the parasite are known to induce NOS, such as glycophosphatidynositol. Alternatively, hemozoin contributes to this process [131].

It has also been proposed that hemozoin causes functional changes in malaria vectors, since the mosquito can ingest inordinate amounts of host blood during the acute phase. Akman-Anderson *et al.*[131] demonstrated that hemozoin can also induce gene expression of NOS in *A. stephensi* and *A. gambiae* cells *in vitro* and in *A. stephensi* tissue *in vivo*. It is also known that the mechanism of NO induction in the midgut of mosquito *A. stephensis* is mediated by NO induction mainly via glycophosphatidynositol, which, despite exerting insulin-like signaling, is not insulinomimetric, requiring AKT/PKB and an ERK activation [103].

Furthermore, GSH is the most abundant antioxidant thiol compound in most cell compartments. However, the *A. gambiae* mosquito lacks the gene to encode the respective sequences of amino acids. Nevertheless, the mosquito synthesizes an enzyme that is able to mimic glutathione actions: TrxR, more specifically Trx-1 [132].

Moreover, vectors have antioxidant molecules to protect cells from oxidative damage as well. Wongtrakul *et al*. [133] identified three isoenzymes for glutathione transferase, an enzyme involved in GSH synthesis, in *Anopheles cracens*, an important malaria vector in Thailand.

Therefore, by using a specific, and probably efficient, oxidative machine, it is quite possible that there are mosquito species resistant to parasite infection, and that this resistance is mediated by different patterns of redox response.

## 5. Antimalarial Drugs and Oxidative Stress

Quinine was among the pioneer antimalarial drugs. It is extracted from the *Cinchona* genus tree or shrub bark in the tropical region of South America. Although the active mechanism of quinine is still not understood well, and despite being used for over 100 years, it is commonly believed to interfere with DNA replication of *Plasmodium*. Quinine was one of the first antimalarial drugs widely used to control the disease, but has fallen into disuse owing to emerging parasite strains resistant to the drug. Consequently, its use has been replaced by more effective synthetic drugs derived from the acridine and quinoline structure, such as chloroquine and mefloquine, aimed at inhibiting heme polymerase and preventing the polymerization of heme to hemozoin, thereby causing oxidative-metabolic effects on the parasite, since iron from the heme group can catalyze reactions that generate free radicals [133] and primaquine, which destroys the gametocytes of malaria parasites.

The pharmacological therapy currently used is based on the susceptibility of the genus *Plasmodium* to free radicals and oxidants, as well as the interference or inhibition of a metabolic synthesis pathway of a molecule essential to the parasite [134].

In fact, several substances used as antimalarials are pro-oxidants, which is why they have pharmacological power. This is the case for chloroquine, primaquine, and artemisinin among others. This effect may be due to the drug’s ability to promote direct production of free radicals [135] or by by inhibiting molecules with antioxidant activity [136].

The *Artemisia annua* plant (*Artemisia*) is known to be the most ancient antimalarial treatment, having been used in China for over 2000 years. It contains artemisinin, a substance which eliminates the blood-stage parasites more rapidly than any other drug and works well against *Plasmodium falciparum* species that are resistant to other drugs. This drug produces free radicals in contact with iron, a common metal in the body, especially within erythrocytes [134]. This mechanism is extremely effective in the destruction process of parasites and causes minimal adverse effects to the host.

Treatment with artemisinin can provide rapid recovery and leads to elimination of parasites, but the reappearance of parasitemia is frequent, which can be explained by the low half-life elimination time of the drug (t_1/2_ = 2, 6 h) and by the decrease of plasma concentrations after repeated doses [137]. Several studies have demonstrated the involvement of oxidative stress in the mechanism of action of artemisinin [138–141].

In pregnant women with the disease, elevated levels of lipid peroxidation markers and reduced ascorbate/glutathione against non-infected pregnant women were noted. In addition, in women treated with antimalarial drugs, lipid peroxidation levels were even higher, with a more intense GSH and ascorbate decrease than in women not treated with these drugs [142].

Also, some associations were tested: association metalloporphyrins/artemisinin [143] and antimalarial/oxidizing reagents that act synergistically [144]. It is worth mentioning that metalloporphyrins/artemisinin effectively act on strains of *P. falciparum* resistant to chloroquine.

Pyrimethamine is another antimalarial drug, which increases the expression of antioxidant enzymes and nitric oxide levels in mice infected with *P. yoelli*, also inducing lipid peroxidation and protein carbonylation in these animals [145].

Moreover, the parasite’s ability to express antioxidant proteins is suggested to be one of the resistance mechanisms to antimalarials, since early transcriptional response of genes involved in antioxidant protein expression confers the adaptive capacity to certain antimalarial drugs [146].

Other pro-oxidant treatment strategies include alternative therapies with antifungal agents such as clotrimazole, based on their ability to inhibit hemo-peroxidase with consequent oxidative stress induction [147].

## 6. Potential Benefit of Adjuvant Antioxidant Therapy for Malaria

Despite the common belief that the ability to induce oxidative stress is a typical active mechanism of antimalarial drugs, in recent years several plant extracts and other natural products have been tested for their antioxidant properties, thus interfering with the mechanisms of the disease by modulating the cellular signaling pathway and not by directly inducing the parasites to death. This approach has shown very promising results, with high rates of schizonticide and antiparasitic activity, but with minor changes in the host redox balance. Some of the plants tested for this purpose include *Piper betle* L. leaves [148], *Anogeissus leiocarpus*[149]*Nigella sativa* seeds [150] and flavonoids from *Artemisia annua* L. [151]. The ability of cocoa fruit to kill malaria parasites is also suggested [152].

Likewise, *Agaricus sylvaticus* mushroom, which exhibits high antioxidant capacity [153], has been tested in mice infected with *P. berghei*. It promotes an increase in the total antioxidant capacity of animals and a decrease in lipid peroxidation and nitric oxide markers in lung and brain samples of these animals. These biochemical changes were correlated with a significant reduction in the parasitemia of animals [17,18].

Furthermore, the use of antioxidant supplements can reverse or minimize the oxidative damage to hosts caused by the use of antimalarial drugs. The administration of curcumin, an herbal antioxidant obtained from *Curcuma longa*, prevented hepatotoxicity in rats treated with chloroquine [154]. Likewise, the administration of glutathione promoted reduced parasitemia and increased survival of mice infected with *P. berghei*[155].

## 7. Conclusions

The role of oxidative stress in the pathophysiology of malaria is a multifactorial phenomenon and represents an important aspect of the intricate and complex host-parasite relationship. The sources of oxygen-nitrogen reactive species generation implicated in the disease are: (1) The host’s inflammatory response; (2) catalysis of Haber-Weiss and Fenton reactions due to high availability of free iron; (3) the occurrence of ischemia and reperfusion syndrome, the fluctuating result in the ability of red blood cells being able to transport oxygen during malarial paroxysm and of cytoadherence; (4) direct production by parasites; and (5) the action of certain pro-oxidants antimalarial drugs.

Therefore, the use of antioxidant supplements of synthetic or natural origin may constitute a far more effective adjuvant antimalarial strategy that causes less damage to the host. However, further research is needed to confirm these suggestions.
